# Each discipline is different: teacher capabilities for future-focussed digitally infused undergraduate programmes

**DOI:** 10.1007/s11423-023-10196-2

**Published:** 2023-02-13

**Authors:** Louise Starkey, Anne Yates, Mairead de Roiste, Karsten Lundqvist, Adreanne Ormond, John Randal, Allan Sylvester

**Affiliations:** 1 School of Education, Victoria University of Wellington, Wellington, New Zealand; 2 School of Geography, Environment and Earth Sciences, Victoria University of Wellington, Wellington, New Zealand; 3 School of Engineering and Computer Science, Victoria University of Wellington, Wellington, New Zealand; 4 School of Economics and Finance, Victoria University of Wellington, Wellington, New Zealand; 5 School of Information Management, Victoria University of Wellington, Wellington, New Zealand

**Keywords:** Disciplinary culture, Higher education, Digital Age, Technology infusion

## Abstract

Disciplines in Higher Education have their own interpretations of what is essential knowledge that influences what is taught, how teaching occurs, and the role of digital tools. Disciplinary culture is dynamic and evolving, informed by disciplinary research and technology improvement. During the COVID-19 pandemic, digital solutions enabled ongoing teaching when undergraduate courses could not be taught on campus, in lecture theatres, seminar rooms, laboratories, or in the field. Using digital tools and changes in teaching practices has created a context where Higher Education teachers must consider how future learning and teaching should occur. To explore this, a cross-discipline team used appreciative inquiry framed in complexity theory to examine how teaching in undergraduate programmes is changing in the digital age and implications for Higher Education teachers. The research identifies how digital technologies influence undergraduate programmes in Applied Statistics, Computer Science, Critical Indigenous Studies, Geography, and Information Systems. Analysis of the case studies identified how disciplinary culture, context, and technology combine to influence pedagogical practice and digital capabilities needed to teach in undergraduate programmes. We conclude that Higher Education teachers require capability in appropriate pedagogical practice that aligns with disciplinary culture and the technologies available.

## Background

University learning is traditionally discipline based. Four broad disciplinary groupings have been identified based on subject matter (Biglan, 1973) and inquiry method (Kolb, 1981). The intellectual clusters are physical sciences, humanities and social sciences, science-based professions and social professions. Within the clusters are distinct disciplines and groupings of professional communities with overlap where each discipline has a culture with common ideas, history and ways of doing things that enable members of the community to identify as belonging (Becher, 1994) and loyal to the discipline area (Healey, 2000) Undergraduate courses provide the context, concepts and inquiry methods for each scholarly generation to adopt the culture and belong to that community.

### Disciplinary differences

An academic discipline consists of a community of scholars, a tradition and history of inquiry that guides data collection and analysis, an epistemological view of what constitutes new knowledge, and ways of communicating across the network of scholars (Davies & Devlin, 2010). These features differ among disciplines, for example, research in science disciplines may use hypothesis testing and quantitative experimental methods to determine a ‘truth’, a social scientist may use qualitative research to develop understanding of a phenomenon, and arts may emphasise the creation of an original artefact. Each discipline has uniqueness in what is taught that includes differing emphasis on the teaching of where disciplinary knowledge comes from or contemporary understanding (Neumann, 2001). What is taught in a discipline influences how teaching occurs and the capabilities needed by teachers in that discipline.

Disciplinary cultures influence practices and beliefs about how teaching and learning occurs and how teaching methods are influenced by beliefs about what it means to be a member of the community. This includes beliefs about how autonomous and collaborative skills are developed (Stark et al., 1990) such as intercultural communication skills in a language, or Socratic argument method in law. The disciplinary communication language may be codified or literary, which may require mastery of skills with highly structured teaching (Smeby, 1996) or flexible, contextual learning tasks. The theory/practice intersection varies between disciplines (Neumann, 2001) with differing levels of critical understanding or practical knowledge emphasised (Movahhed, 2021) which influences academics’ beliefs about what and how skills should be taught and resourcing needs such as physical spaces, equipment and access to expertise (Stark et al., 1990). Assessment practices and priorities follow discipline specific cultures, for example final examinations, essays, oral presentations, or practical examinations (Neumann, 2001). The disciplinary beliefs and traditions (culture) strongly influence teaching practices but are not the only influences.

### Digital technologies in the disciplines

Digital technologies are embedded in universities; they are embedded across Higher Education teachers’ work including research, teaching, administration and networking, with each function of digital technologies requiring requisite capabilities of higher education teachers. The academic discipline frames a culture that influences how technology is used for teaching and learning (Shelton, 2014) and the choice of digital tools can differ between disciplines (Czerniewicz & Brown, 2007). Use can vary, with Soft and Applied disciplines found to be more digitally orientated (Lam et al., 2014). In addition, the perceived usefulness of generic educational technology can vary between disciplines, for example, lecture recording technology has been perceived as more beneficial in social sciences than natural sciences due to differing pedagogical practices and expectations of students (Dona et al., 2017). However, limited research explores or compares disciplinary cultures in the digital age, the implications for teaching practice and the requisite teacher capabilities required.

### Post-COVID teacher capabilities

During the COVID-19 pandemic, teaching in higher education moved to online. Travel was restricted and field work cancelled, or digital alternatives explored. This sudden change increased the use of digital tools for teaching and learning, particularly learning management systems (LMS) and video-conferencing platforms such as Zoom, whether teachers possessed the capabilities to incorporate online learning or not.

Pre-pandemic research developed a range of generic models or frameworks, of which some categorise the knowledge or competencies required by teachers to teach in technology enhanced learning environments, such as the TPaCK framework (Koehler & Mishra, 2009) and the European digital competence framework for educators (Caena & Redecker, 2019). These models and those that identify factors that influence successful technology integration and usage by teachers (for example, Tondeur et al., 2021) and professional development frameworks, such as Philipsen et al., (2019), were developed for the schooling sector and do not include disciplinary differences. As universities consider post-COVID education, we consider how digital technologies influence teaching within different disciplines and the implications for Higher Education teacher capabilities in the future. This interdisciplinary discussion aimed to examine:

1. How are digital technologies influencing culture of the disciplines?
2. How are digital technologies changing undergraduate teaching and learning in specific disciplines?
3. What are the implications for Higher Education teacher capabilities?

Educational systems, institutions and practices are dynamic and emergent, operating in unpredictable and changing external environments (Morrison, 2012). Therefore, we used a complexity thinking approach to examine how digital technologies are impacting the culture of disciplines and the capabilities required by teachers within those disciplines. Complexity theory focuses on the study of complex systems and how they behave and change (Davis & Sumara, 2014). A complex system is one with many interconnecting components interacting with each other and these components change, learn, and adapt. A complexity thinking approach was used as this research focuses on five academic fields, how they have changed in the digital age and the ensuant capabilities needed by teachers in those disciplines.

### Research method

**Fig. 1:**
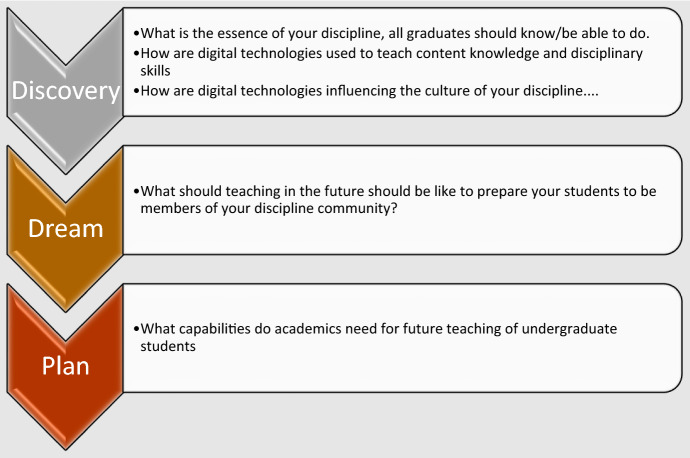
Appreciative inquiry framing

Our research is framed within the beginning stages of an appreciative inquiry. An appreciative inquiry aims to collaboratively identify and share what works well in practice, rather than trying to fix what doesn’t work; appreciative inquiry offers an affirmative approach for future initiatives (Shuayb et al., 2009). Appreciative inquiry has five phases of which we have used the first three to frame the research (Fig. 1). The first phase is discovery, identifying key aspects of different disciplinary contexts. The second phase, to dream, is when we consider how each discipline could be in the future. The third phase is to plan what digital capabilities the academic staff need to teach in the digital age and what resources and conditions would allow this to happen.

Appreciative Inquiry is a participatory, collaborative approach to research that employs techniques such as group discussion and interviews (Shuayb et al. 2009). In this project seven Higher Education teachers, from one university in New Zealand:

- met twice for group discussion (meetings were each of two hours duration) – October 2021.
- individually reflected in writing on the research questions – October-February 2022.
- Discussed with a lead researcher to ensure their reflections complied with the research questions -January-February 2022.

The process was led by two Education faculty academics who purposively selected five academics from each of the disciplinary cultures identified in the literature. The disciplinary experts were selected because they were known to have an interest in teaching and the future of teaching, and because they taught an undergraduate course in their discipline. In accordance with a participatory approach the five disciplinary academics were participants and members of the research team. At the first meeting, the seven-member team discussed the project and research questions, and boundaries of the discovery and dreaming phases of an appreciative inquiry. Following this, the five discipline academics individually reflected in writing on teacher capabilities needed to use digital technologies in higher education and in their disciplinary field, in particular opportunities and challenges presented by Covid-19 remote teaching and learning, and possibilities for future learning designs. They also considered the impact of the digital age on teaching in undergraduate programmes. They individually wrote their reflections as five case studies because according to Hetherington (2013), a case study approach is particularly suitable for research framed by complexity thinking because complexity is contextual. The written case studies were shared among the group and a lead researcher then met with each of the five academics, for approximately one hour, to ensure the reflections were in accordance with the research questions. The revised reflections were shared with all researchers before a second team meeting where cross-case themes were developed through discussion and agreement, and the planning phase (teacher capabilities needed) discussed. Abductive reasoning was used to develop the cross-case themes, because this type of analysis starts with observations, that may be incomplete, and then seeks the most likely explanation for the phenomena observed (Ong, 2012).

### Findings

The findings, written by the disciplinary experts, outline each of the disciplines in terms of the essence of the discipline, how digital technologies are changing undergraduate teaching and learning and the implications for Higher Education teacher capabilities.

### Information systems

Information systems (IS) is a relatively new discipline, stemming from management, psychology, computer science and information science disciplines in the 1960s and 70s and became a recognisable discipline in the late 1970’s (Rai, 2016). The discipline focuses on the intersection of human activity relationship with data, information and technology artefacts, organizational processes, the creation, adoption, and use of technologies, and the impacts of technology on individuals and society.

#### Culture of the discipline

Education in IS at the undergraduate level is broad, the discipline covers topics such as: Information Technology systems architecture, business ethics, organizational strategy and governance, data and content management, systems design and development, and innovation (Topi et al., 2019). The competency areas are reviewed routinely by working groups within the Association for Information Systems (AIS) and the Association for Computing Machinery (ACM). More broadly, the aim of undergraduate IS education is for students to understand the relationship of people and communities with information, and how that information is created, used, and moved around. Information management as an applied discipline develops practical skills by using programming and modelling tools to build the foundation skills of information systems practice, understand how the related technologies work together, and apply the vocabulary of the systems development.

#### Teaching IS in the digital age

Using digital technology for teaching and teaching about digital technology is not synonymous. Teaching in IS presents some interesting challenges. The topics are about digital technologies, in many settings the teaching format relies on those same technologies so the opportunity to model the phenomena is sometimes useful but can also complicate learning when students use systems that necessarily mask complexity. Information Systems is introduced to students at university and needs contextualization by using relevant case examples. First year IS courses have over 650 students twice per year taught through traditional structures of lectures, tutorials, and practical workshops. Lectures lend themselves to delivering content which can be transferred to online dissemination via short topic videos and exemplars. The tutorial structure requires differentiation from lecture topics and uses constructivist approaches to cater for learners at diverse conceptual stages.

IS evolves continually in the digital age because technology and use of tools change rapidly. Students joining undergraduate programmes have experience of using digital technologies, but the myth that millennials understand technology intuitively is not the case (Kirschner et al., 2017). As an applied subject, assessment in IS includes evaluating conceptual and practical knowledge. Students learn by applying understanding in a laboratory situation to code and create online artefacts that manipulate data, apply logic through algorithmic thinking and communicate ideas using digital technologies. Coding on its own is not sufficient, students need to develop a range of information skills including interviewing for requirements, process modelling working collaborative to develop and present design ideas.

Digital technologies, while inherent in the IS discipline, create an opportunity to refactor how the discipline is taught. Video and multimedia provide the opportunity to personalize and offer relevant case studies to enable students to engage with and understand threshold concepts being taught. The use of digital delivery opens the path to new ways of learning where student agency and autonomy can be leveraged by providing students with the means to self-curate the sequence and depth of engagement specific to their own needs.

#### Teacher capabilities for the digital context

Teachers in IS need to possess the digital capabilities of the discipline, they need the range of digital capabilities that are taught to students. These include coding, creating online artefacts, applying logic through algorithmic thinking, communicating ideas through digital technologies. interviewing clients for requirements, process modelling, working collaboratively to develop and present design ideas.

In addition to discipline specific digital capabilities teachers need generic digital and pedagogical skills that will allow undergraduate IS courses in the future to change. IS teachers need generic digital capabilities to create and curate resources that will allow flipped classrooms, online laboratories, and digitally enabled internships. They also need digital, pedagogical skills that enable constructivist approaches to differentiation in online tutorials to cater for learners at diverse conceptual stages This creating of new learning pathways and guidance for students will involve challenging many of the existing norms of university teaching such as the notion of a course, progression neatly from novice to advanced learner and the cycle of refreshing content that learning in a digital age requires. Digital, pedagogical capabilities and up to date discipline knowledge are key in this subject.

### Geography

Geography is the study of places and people and the complex and dynamic physical, environmental, and social relationships between them. Within the discipline, three key strands exist: Physical Geography, Human Geography, and GIS (Geographic Information Science). Physical Geography explores the geomorphic processes that shape the Earth’s surface, such as natural hazards, climate change, and hydrology. Human Geography examines how human cultures impact and are impacted by their interactions with place or places, such as globalisation, sustainability, and social justice. GIS is the study of the capture, processing, analysis and visualization of spatial and geographic data using computer-based systems such as digital mapping.

#### Culture of the discipline

Each of the three strands invoke different teaching methods and content in undergraduate programmes. Physical Geography has a strong emphasis on learning through experience in the laboratory and field as well as being taught through lectures and tutorials. It generally follows a postpositivist approach favouring quantitative methods and empirical observation and measurement. Human Geography primarily follows a face-to-face lecture and tutorial model where field teaching forms a smaller but important component. Approaches commonly span epistemologies from transformative with activist academics to pragmatism often favouring mixed methods and applied research. GIS is taught through face-to-face lectures and computer laboratory sessions. It is predominantly postpositivist, but critical GIS approaches also favour mixed methods and the incorporation of qualitative data. This mix of approaches across the strands exposes students to a diversity of thinking and supports an understanding of different ways of knowledge building.

An important aspect across the strands is practical learning in the ‘field’. Field teaching “is one of the most powerful learning invitations in the toolkit of Geographical Education” (France & Haigh, 2018, p. 498). In the field, students learn by doing through recording scientific measurements (e.g., measuring stream channel depth or discharge), observation (such as foot traffic counts or noting building density), or participant surveys. These real-world experiences are valuable in the “locus of becoming for the real geographer” (Powell, 2002, p267).

#### Teaching Geography in the digital age

The implementation of digital tools has caused the discipline of Geography to adapt, altering aspects of disciplinary culture. GIS is increasingly viewed as a fundamental skill set of the discipline. Undergraduate students learn to map digital data and combine disparate datasets to build an understanding of different areas of enquiry from hazards to cultural mapping. Their learning can be driven by a software-oriented approach or by taking a wider view, looking at the software tools in the context of concepts, techniques and approaches behind the use of the software (e.g. data sovereignty). The use of virtual reality (VR) enables fieldwork and laboratory teaching without having to be physically present in a field location. Specialist digital equipment has been developed that has broadened the experience of students learning in the laboratory or for fieldwork. For example, UAVs (Uncrewed Aerial Vehicles) enable land cover capture or the creation of elevation models for hydrological or other landscape modelling.

However, such resources are expensive to develop and can be limited by resolution and digital storage constraints. Field work is place-based teaching and digital replication requires the development of substantial digital resources for individual locations specific to courses that mimic complex physical environments and the tactile nature of experiential learning. Further, soft skills, such as teamwork and communication skills, and the creation of a disciplinary identity are often enhanced by fieldwork, and these are difficult to replicate in an online environment with meaningful social interactions and authentic practical experiences.

#### Teacher capabilities

A range of digital tools are embedded within Geography undergraduate programmes. Generic educational digital technologies used for learning and teaching, and communication, such as the learning management system, and communications tools, such as Zoom, align with lecture or tutorial type teaching. Teachers in this field need to master the use of this range of generic digital tools and knowledge of how to teach in the evolving post Covid context. In addition, to generic digital capabilities, Higher Education Geography teachers primarily within Physical Geography and GIS need capabilities in discipline specific applications such as GIS, Remote Sensing software, and UAVs, which are increasingly considered fundamental skill sets of the discipline.

### Statistics for business

#### Culture of the discipline

While a field in its own right, statistics courses within the field of business are common-place. It is a subject with mathematical underpinnings but does not require those learning or applying statistical methods to be mathematicians. It requires students to understand statistical concepts as they are applied to business contexts with the focus firmly on mastering the application of statistical skills. Therefore, the discipline lends itself to mastery focused pedagogical practices (Parker & Roumell, 2020). Such practices scaffold students through a learning progression from novice to mastery with assessment and feedback integral to the process.

#### Teaching statistics for business in the digital age

Digital technologies have changed pedagogical practices and assessment in applied statistics. Last century, calculators and Eton’s tables were ubiquitous, and the focus on arithmetic both in teaching and subsequent assessment was necessary. Assignments were handwritten, and tests and examinations constrained to very small data sets with manual calculations. This changed when students gained access to cheap digital devices capable of running statistical software, and free but powerful statistical tools, many built on the R programming language, became available. The digital technology allowed automated statistical analysis with larger and more authentic datasets.

Prior to Covid-19, digital applications were used in assessment practices. Assignments were submitted and given feedback through an online learning management system (LMS), and featured written components, digital artefacts from spreadsheets and specialist software taught in the course. Tests of knowledge and skills were invigilated in computer laboratories through a mix of multichoice questions, numerical input based on use of the software tools, and manually marked short answer questions. This was structurally constraining as laboratories have limited capacity, requiring multiple scheduled assessment times.

The Covid-19 pandemic changed teaching in the course, freeing some constraints around assessment and enabling structural flexibility for how students can access learning. Because remote learners could not easily be invigilated at scale, all assessment became uninvigilated. Students now do major tests on the device on which they learn, which presumably helps with confidence and performance, and greater flexibility in the timing of the assessment has enabled students to manage their workload to a greater extent than was previously possible. Students using their own devices and testing environment overcomes pressure on the university’s physical resources, however, it moves the pressure onto individual students and assumes access to a device.

As the course became free from the constraints of synchronous assessment the teaching evolved further. The use of software and teaching videos now guide student learning within a LMS and targeted skills videos are shared through YouTube which enable student access beyond the course. This has resulted in limited need for scheduled face-to-face classes, ‘lectures’ have become scheduled Question and Answer sessions and ‘tutorials’ have been replaced by an online help desk where students. The knowledge/theory can be presented through interactive multimedia content drawing on different contextual examples to enable personalisation and engagement by the students depending on their interests, experiences and perspective (culturally appropriate examples).

Applying approximations of practice (Grossman et al., 2009) enables students to gain mastery of the skills by observing, then practicing with similar data, moving further from the example to mastery where students apply statistical analysis in different contexts. The digital tools enable students to do this in their own time, at their own pace using examples with Artificial Intelligence (AI) providing immediate feedback and differing next steps. This is a significantly different way of teaching to one in which Higher Education teachers present knowledge then test students on their use of this knowledge.

#### Teacher capabilities

Higher Education teachers of applied statistics for business teaching in a student-centric way require not only strong content knowledge of the digital applications inherent in the subject, but also generic digital capabilities to develop and curate multimedia and interactive resources, and the ability to contextualise case studies. They also require pedagogical knowledge to design differentiated learning pathways for students and knowledge of how to design digital learning activities that will engage students. Higher Education teachers are crucial in the development of digitally enhanced, course specific support for students learning in this flexible way.

### Critical indigenous studies

#### Culture of the discipline

Critical Indigenous Studies (Hokowhitu et al., 2021) is a field that has emerged from the worldviews and knowledge systems of the earliest known native, indigenous and First peoples to inhabit the Earth. It is an interdisciplinary field with the knowledge traditions reflecting the diverse geographies, spatial and temporal aspects, and historical political relationships that underpin and create indigenous community. The natural world ecologies, which are often referred to as ‘place’ (Tuck & Mckenzie, 2015), that are central to many indigenous communities are differentiated by significant natural and material world reference points so that each community and place is unique. Threaded within their distinctiveness is a relationality or interrelationship with the non-human world to co-produce holistic ways of being and living. The human and non-human world are connected in ways that co-constitute ecologies of knowledge (Santos, 2007). In this regard, the field of Critical Indigenous Studies is a vanguard of unique interrelations from which a pluriverse (Santos, 2014) of human, natural and material world intelligence, energy, and agency (Barad, 2007) is presented.

Within the Westernised university, a priority of Critical Indigenous Studies is the preservation of Indigenous peoples through self-determination and cultural revitalisation, and resurgence (Alfred & Corntassle, 2005). Critical Indigenous Studies does this through the indigenous political agenda, known as decolonisation (Smith, 2021) that is shaped by the different needs of the communities across the globe.

Within Aotearoa/New Zealand one way decolonisation has occurred within the Westernised university is through the introduction of knowledge systems and traditions of the Indigenous Māori people. The knowledge of Māori, within the university and many other institutions has come to be referred to as matauranga Māori (Smith et al., 2016) or Kaupapa Māori (Pihama et al., 2015). Both cultural frameworks speak to research methodologies, ethics, teaching and learning, and course content and offer an Indigenous standpoint to rebalance the Cartesian philosophy that gave rational attainment of mind over body or rational attainment of human over non-human in ways that excluded Māori knowledge systems. A fundamental requirement of teaching indigenous Māori knowledge is an understanding of Te ao Māori or the Māori world. This usually requires Māori academics who are masterful in Te ao Māori as well as Western competencies and can weave within and between the two worldviews.

#### Teaching critical indigenous studies in the digital age

The online learning environments used in universities have evolved within a western educational paradigm and are largely orientated towards a Eurocentric knowledge system. For Critical Indigenous Studies (CIS), this means that the holistic knowledge systems and linguistically diverse communities we draw from continue to be marginalised. With the development of digital technology largely being drawn from non-indigenous thought the distribution of digital and technological advancement once again is positioned largely outside of the indigenous world. Therefore, the question of how digital technologies are changing CIS might be addressed by considering the ways indigenous communities are harnessing digital technology to build solidarity, stimulate transformation and operationalise decolonisation.

One way this has occurred is through digital technology being used so the diverse knowledge of people and entities within communities are more accessible. Through technology, communication within and between different communities has become less complex, creating more options, with swifter dissemination to a wider audience possible. For example, remote areas that were unreachable and therefore unteachable are now reachable through digital technology. Knowledge holders, such as elders, can share their life wisdom, understanding of cultural practices and expertise from their homes, community and ancestral landscape. Through digital technology media they can witness to the relational connection between humanity and the natural world they might know of from their life experience and the gifts it can offer humanity. This kind of culturally relevant, living testimony grounded in context and intimately connected to the community that has produced it, is known as place-based education and is vital to CIS. In this way digital technologies have allowed for increased sharing of strategies of survivance, practices of solidarity and mobilisation of international community.

One of the historical hangovers of colonial erasure for indigenous people engaging with an institution such as university is cultural alienation, and isolation which can lead to disengagement and eventual withdrawal. For CIS, face-to-face interactions established relationships that buffered issues of this kind helping to create community and maintain student engagement. With the shift to emergency remote teaching due to Covid-19, teacher to student relationships were altered, with many experiencing digital isolation (Bennett et al., 2020). Grappling with these changes, CIS teachers sought to maintain student engagement by using digital technology to explore critical wicked problems. Issues that include the impact of the pandemic upon indigenous people, health inequities, access to resources and advocacy for indigenous needs. Technology such as podcasts, video, Zoom and Facebook have allowed for a flow of information and visual documentation direct from the coalface of lived reality into online classrooms and have given lively substance to student’s thoughts and lessons in ways that traditional lectures have lacked. Relationships have been enhanced through digital technology, with scholars from the institution and community able to participate in online classes and help shape students thought with their life experience, content expertise and scholarly activism.

#### Teacher capabilities

For many, building teacher digital capabilities in terms of responding to indigenous peoples and an indigenous educational agenda is not a simple issue. It is complicated because a Westernized university is based on knowledge domination and does not allow for a plurality of knowledges so indigenous thought might flourish. CIS in the digital age requires educators that are grounded in an anti-colonial stance and who uphold decolonisation through embracing indigenous knowledge and ways of being. Therefore, teachers must understand the political nature of CIS and be culturally capable in ways that inform their pedagogical practice. Teachers in CIS need generic technical capabilities of using, creating and curating digital artefacts and creating online learning spaces for their students. But in addition, they need capabilities in indigenous knowledge and worldviews which includes selecting digital tools and pedagogical approaches which employ appropriate cultural norms and codes of written or spoken communication when giving feedback to students (Istenič, 2021), using technologies such as Zoom to engage students with community experts and design community driven collaboration.

### Computer science

#### Culture of the discipline

Computer Science (CS) grew out of mathematics in the efforts of developing automatic general-purpose computers (Haigh, 2014). Consequently, traditions of the field were initially influenced by mathematics and other sciences. However, as CS has grown into a more applied field, it is increasingly cross-disciplinary resulting in embracing other disciplines’ methodologies and cultures with both qualitative and quantitative inquiry prominent and accepted (Belford et al., 2020). This evolution is changing undergraduate study of CS. The Association of Computing Machinery (ACM), which develops curriculum recommendations for the field, modified the principles behind the curriculum design in 2013 to embrace a “Big Tent” view of CS to develop a discipline that works with and integrates into other disciplines (Joint Taskforce, 2013). Within this flexibility undergraduate students develop both technical and theoretical understanding of the field and apply this knowledge to collaboratively solve programming problems.

CS is a popular applied degree with many job opportunities for graduates. Programming is an important skill within other subjects; therefore, it is common to have large class sizes in the first-year courses and face-to-face lecture-based teaching. This is problematic because developing practical skills is difficult in lecture-based, teacher-centred teaching modalities. Consequently, laboratories or tutorials are used to supplement the lectures, led by undergraduate teaching assistants who use activities and programming assignments (Barak et al., 2007). More practical, multi-modal, constructivist teaching methods are used at a minority of institutions (Falkner & Falkner, 2012).

Assessment is typically through assignments and exams. These are commonly individual, but due to the collaborative nature of the field, there are also group collaborative exercises. Plagiarism is currently a subject of particular concern for the area when assessing technical skills (Albluwi, 2019) because online resources are readily available, including tools that generate undetectable copies of code and Artificial Intelligence (AI)-supported programming aids (Pearce et al., 2021). The field of CS reuses source code and relies on shared contribution, so it is difficult to define plagiarism precisely when considering programming assessments.

#### Teaching CS in the digital age

Digital technologies exist because of Computer Science, and Computer Science exists because of digital technologies. Consequently, the discipline has been a significant contributor in facilitating and ‘driving’ the digital age. The pace of change in the field can be problematic, even to the degree that tools students were taught to use in their first year of study might be outdated when they graduate. Therefore, undergraduate study focuses on the fundamental ideas or concepts of Computer Science and how these skills are transferable (Joint task force, 2013).

The introduction of AI programming tools, such as Copilot, means that the concept of plagiarism is fundamentally changing (Pearse et al., 2021). In a world where a robot can write code to solve non-trivial problems, without a way of knowing it originated from a robot, it does not make sense to ask students to write code using trivial problems. When a code generator will generate perfect code, the emphasis should be on understanding what the code does, what high-quality code is, and why two different solutions are not similarly good.

Most concepts within CS and software engineering are suitable to be taught online and working remotely is common practice within the CS industry. Tools such as videoconferencing, simulations, screen-sharing and online collaborative tools enable online teaching and mentoring. The industry uses tools and methods to collaborate via the internet, therefore collaborating and working online is part of the work of being a computer scientist. However, programming teamwork skills are challenging to teach (Sancho-Thomas et al., 2009) and especially online where differences in programming proficiency and social skills can negatively influence the learning (Konak et al., 2018). Currently, Higher Education teachers seem anxious about integrating online industrial collaborative tools, possibly because they are concerned about plagiarism. However, the IT industry is increasingly more interested in graduates with high levels of collaborative and social skills (Stevens & Norman, 2016).

#### Teacher capabilities

Computer Science academics must have a strong knowledge and capabilities in the digital tools and programming software used within the discipline, and the fast paced-nature of change requires constant upskilling in technical aspects of the discipline. Advances in the discipline also require changes in pedagogical paradigms, for example, the introduction of AI programming means teachers cannot rely on asking students to solve programming problems that AI can do, instead they need to focus on computational thinking and computer science theories when teaching and assessing students – requiring requisite pedagogical and technological capabilities. In addition, teachers of CS need capabilities in the effective of use digital, educational technology for teaching and learning. For example, they need to be able to develop capabilities to integrate new digital tools and ways of learning into undergraduate education, such as student collaboration and co-development, but these skills cannot be assumed.

## Discussion

The infusion of digital technologies within Higher Education teaching is changing disciplinary undergraduate curriculum, pedagogies and teachers’ digital capabilities. The disciplinary approach has highlighted that digital technology is not only integrated into teaching and learning practices, but the disciplines themselves are evolving as digital technology develops.

### Changing cultures

The academics described ongoing integration of digital technology into their discipline and the resultant change in their disciplines and capabilities needed by teachers. These descriptions confirm that disciplines are not static, they evolve over time, and there are multiple influences on change which reflects the complexity of educational change (Davis & Sumara, 2014). The disciplines reflect changes attributable to the digital technology available and the broader political and social context (e.g. Covid 19) that have hastened the adoption of digital technologies in higher education.

For Information Systems (IS) and Computer Science (CS) digital technology is central. IS as a modern discipline developed alongside the technology and CS exists because of computers, or computers exist because of CS. These disciplines have emerged because of the digital age. The undergraduate curriculum in these disciplines evolves quickly as technology changes and with CS, the field creates the changes. Computer technology is integral to the culture of CS and IS. While Higher Education teachers in these disciplines have inherently high digital capability, they are often highly capable in focused areas but may lack skill in education-oriented digital tool sets, pedagogical flexibility, and changes in the disciplines require different pedagogical approaches.

Statistics and Geography existed long before the digital age. Digital technology has been superimposed into these existing disciplines, changing the digital capabilities required by teachers, methods and content taught at undergraduate level. For example, GIS has added a new strand to Geography and statistical programmes have enabled computer assisted analysis with much larger datasets in Statistics. Digital technology hasn’t necessarily changed the conceptual knowledge taught in Statistics but has allowed different ways for the discipline to be operationalised. The discipline is now focused on teaching software use to carry out statistical operations, rather than learning complex formulae to carry out manual calculations.

Critical Indigenous Studies is a relatively new discipline that sits outside subject specific digital technology but generic digital technology broadens the field and allows greater inclusion. However, the ability of digital technology to provide a more inclusive teaching space creates a dilemma because the opportunity for greater inclusion by connecting with formerly unreachable communities, can be excluding to those who lack access to the technology.

### Changing pedagogy

Unsurprisingly, each discipline has uniqueness, what is taught in a discipline influences how teaching occurs (Neumann, 2001). While all disciplines followed aspects of a traditional university model of face-to-face lectures and tutorials each also has its discipline specific teaching methods. The Higher Education teachers in this study identified skills-based learning in their discipline which relate to preparing graduates for the workplace which were influenced by the digital age context. This ranged from knowing how to programme computers, using statistical packages to embodied learning (see Stolz 2015) such as fieldwork, critical thinking and relationality. Different modes of learning alter the student experience, and the role and digital capabilities of the teacher.

The Sciences have a strong tradition of field work and ‘real-world’ experiences which are the essence of being a Scientist. Virtual reality (VR) can be used to replicate field work in the physical sciences, but with Geography, it loses the embodied presence of being in a complex natural environment and team-based, problem-solving. Working collaboratively is key in Computer Science and Information Systems. A teacher leading virtual field work or laboratory activities needs the capability to make learning authentic and to teach students how to work collaboratively through the digital medium of instruction (Chatterjee, 2021). This requires knowledge of the discipline culture, the technology being used, and the capability to develop collaboration and authentic contexts in a digital teaching environment.

Authenticity differs between the disciplines. Authenticity in Critical Indigenous Studies includes students engaging with real, societal scenarios (Istenič, 2021), whereas in Computer Science authenticity is solving programming problems and in Science experiencing scientific processes as a scientist would. When digital tools are used to substitute the lived experience such as VR field trips, authenticity can be compromised which has implications for graduates being work-place ready. This can be overcome if approximations of practice are embedded in the programme (see Grossman et al., 2009), scaffolding students from the digital learning experience to the authentic context. This pedagogical approach requires teacher capability that draws on knowledge of discipline culture, pedagogy and digital tools.

Relationality in Critical Indigenous Studies is not the same as teaching students how to collaborate, it is a way of being. Replicating relationality through digital means can be limited through the loss of non-verbal aspects of communication. However, Payne et al., (2022) argue that due to the ongoing necessity of online education and the affordances this can offer, facilitating trust between teachers and students (particularly those from First Nations) is important. Like collaboration, this requires knowledge of relationality from a discipline culture context, capability in the digital technologies and pedagogical practices which build relationality and trust through the online or physical context.

While digital tools can increase flexibility and opportunities, they can also constrain these because the digital technology used in some subjects reduces opportunities for learning. For example, GIS technology in Geography, and learning about robotics, VR, or super-computing in Computer Science currently need to be in a laboratory due to high hardware costs which are prohibitive to individual students.

### Pedagogical paradigm change

Change in discipline culture and the digital tools available can instigate a pedagogical paradigm change (Webb & Cox, 2004). This is seen in Computer Science, Information Systems and Statistics for Business where digital technology enables a personalised mastery approach with a focus on understanding theory or concepts of the discipline. Capabilities teachers require include in-depth knowledge of the discipline, technical skills to develop or refine digital resources that have authentic scaffolded examples, and knowledge of how to coach students within online systems, and ability to facilitate online discussion to develop critical thinking. The Higher Education teachers in these fields saw the potential of digital technologies to provide differentiated learning but recognised the pedagogical and digital capabilities needed differ from those needed for traditional one-size fits all, face-to-face lectures. Changing pedagogical approaches from a traditional face-to-face lecture/tutorial and examination model to more online and blended learning also requires flexibility in the policies and structures of the institution (Lock et al., 2021).

### Context influencing pedagogical change

Changes to discipline areas in the digital age reflect broader societal trends and tensions. This study was carried out after the COVID-19 pandemic Great Online Transition (Howard et al., 2022) in a context where neoliberal political agendas have introduced the massification of higher education and a focus on preparing work-ready graduates (Tight, 2019). At the same time, societal focus on equity and inclusion in education has created an imperative for educators to recognise differences in knowledge and experiences students bring to their study which requires flexibility in courses and pedagogical practices. The traditional university organisational structures built around presenting information through synchronous, face-to-face large lectures have been challenged by the GOT, the imperative to be inclusive of student diversity and the need to ensure students have equitable access to quality learning experiences. For the academics in this study this is creating some uncertainty, but also opening possibilities to exploring pedagogies in a more flexible, digitally infused higher education system. It is also highlighting the need for teacher pedagogical and digital capabilities that can take advantage of the opportunities of increased online and blended learning, such as flexibility in learning, while also having capabilities to address inequities that can occur in online learning.

### Teacher capabilities

The academics in this study identified how disciplinary culture and technology are intertwined with pedagogical approaches and the capacity needed to use digital tools are influenced by the tools and the discipline. Teaching capabilities vary as they are influenced by disciplinary culture and disciplinary specific, as well as generic digital technologies (Czerniewicz & Brown, 2007). All Higher Education teachers in this study cited the need to use generic digital technologies, such as Zoom or video capture, in pedagogically appropriate ways. For example, in Critical Indigenous Studies, it was important to use Zoom to access students in remote areas that were previously unreachable and also knowledge holders, such as elders, to share their wisdom and understanding of cultural practices. For Statistics for Business, Zoom was seen as a way of providing differentiated learning to students with differing learning needs. As well as the technical capabilities to use this technology, the academics emphasised the different pedagogical skills inherent for their disciplines. In Statistics for Business this was the ability to design differentiated learning activities that can be delivered over a digital platform, whereas for CIS capabilities were needed to select digital tools and pedagogical approaches which employ appropriate cultural norms. As with Xue et al., (2021), this highlights that even though teachers may be using the same technology tool, their specific contexts lead to differences in their practices and the need for differing capabilities.

Commonly used technology integration frameworks associated with teacher capabilities or knowledge were developed for the schooling sector. For example, the TPaCK framework (Koehler & Mishra, 2009) was developed to examine technological, pedagogical and content knowledge that schoolteachers require for technology integration. This study has found that in Higher Education, it is the context and disciplinary culture; the traditions, skills and knowledge within a subject that influences pedagogical practices, how digital technology is used, and the capabilities needed to use digital technology within each context. The disciplines of Computer Science, Statistics for Business, Information Systems and Geography required discipline specific digital capabilities, such as coding in computer Science and Information Systems, capabilities in GIS, VR and UAVs for Geography and knowledge of statistics and statistical software in Statistics for Business.

## Conclusion

This study used the first three phases of appreciative inquiry to research how digital technologies are influencing culture of disciplines, how digital technologies are changing undergraduate teaching and learning in specific disciplines and the implications for Higher Education teacher capabilities. The findings concur with previous literature in that the theory/practice intersection varies among disciplines (Neumann, 2001) that have cultural differences in what and how teaching occurs (Movahhed, 2021). The infusion of digital technologies in disciplines and the rethinking of pedagogical practices because of the GOT (Howard et al., 2022) appear to be altering disciplinary cultures, amplifying differences in discipline specific pedagogical approaches and creating the need for varying teacher digital capabilities. Disciplinary culture is the basis of Higher Education teacher identity and underpins pedagogical practice and use of digital technology. This in turn influences the digital capabilities needed by Higher Education teachers. Digital capabilities are both discipline specific and generic, although the use of generic tools differs among the disciplines. The academics in this study identified that discipline specific digital capabilities are developed through being part of that discipline, whereas generic digital capabilities that required pedagogical knowledge were acquired in a more haphazard way with participants acknowledging that not all teachers possessed these. However, acquiring these capabilities is essential to ensuring students have equitable access to quality learning experiences.

This has the potential to challenge organisational structures of institutions as faculties use digital tools to induct undergraduate students into the cultures of the disciplines. It also requires that institutions provide resources to enable these capabilities to develop and as found by Buchholz et al., (2019), time is a key resource in developing new capabilities.

Higher Education teachers require pedagogical capabilities to teach in undergraduate programmes in the digital future. Previous studies that have examined change in the COVID-19 era have predominantly focused on the schooling sector or teaching within a specific discipline. The professional learning models for developing teacher capability need to be adapted for Higher Education and to centre disciplinary culture. A move away from lectures toward greater flexibility in organisational structures and inclusive approaches will mean that Higher Education teachers need to develop capability in appropriate pedagogical practices that align with disciplinary culture and the technologies available.
